# Differential Tissue‐Coupled Powering for Battery‐Free Injectable Electroceuticals

**DOI:** 10.1002/advs.76915

**Published:** 2026-08-05

**Authors:** Han Wu, Sultan Mahmud, Mali Halac, Asif Iftekhar Omi, Domenica Sofia Baez Rodas, Jeremiasz Dados, Chaojun Cheng, Anyu Jiang, Cameron Wallace, Anvi Singh, Erin Patrick, Baibhab Chatterjee, Shriya Srinivasan, Adam Khalifa

**Affiliations:** ^1^ Department of Electrical and Computer Engineering University of Florida Gainesville USA; ^2^ Department of Bioengineering Harvard University Cambridge USA

**Keywords:** battery‐free, electroceuticals, implantable medical devices, injectable devices, tissue coupling

## Abstract

Electroceutical implants that deliver targeted neural stimulation have shown therapeutic potential for a wide range of neurological and peripheral disorders, yet wirelessly powering ultra‐miniaturized, fully injectable systems remains a critical challenge. Here, we report a Thread‐like Injectable Neural TechnologY (TINY) based on differential tissue‐coupled powering (DTCP), which transmits energy through tissue using MHz‐range differential fields generated by a compact, wearable transmitter. DTCP allows power harvesting to scale with implant length rather than cross‐section, enabling a flexible, thread‐like implant that integrates a custom ASIC with PEDOT‐coated receiver and stimulation electrodes. Benchtop experiments in tissue‐mimicking agar phantoms characterize power‐transfer efficiency (PTE) and reveal that PTE increases with implant length while remaining highly tolerant to angular misalignment. In vivo tests in rat hindlimbs further demonstrate wireless sciatic nerve activation through tissue at centimeter‐scale depths, confirming effective transcutaneous energy delivery for neurostimulation. A 20‐day implantation study provides a short‐term/subacute assessment of device positioning and local tissue response. Together, these findings address long‐standing challenges in wirelessly powering injectable electroceuticals and establish DTCP as a scalable and alignment‐robust powering strategy for future minimally invasive neuromodulation therapies.

## Graphical abstract

A thread‐like injectable neural stimulator is powered through differential tissue‐coupled powering (DTCP), which harvests RF‐frequency tissue potentials with an elongated receiver while preserving a injectable cross‐section. The system integrates an ASIC, stimulation electrodes, and a portable transmitter, enabling wireless peripheral nerve stimulation, orientation‐tolerant power delivery, and short‐term in vivo functional validation.

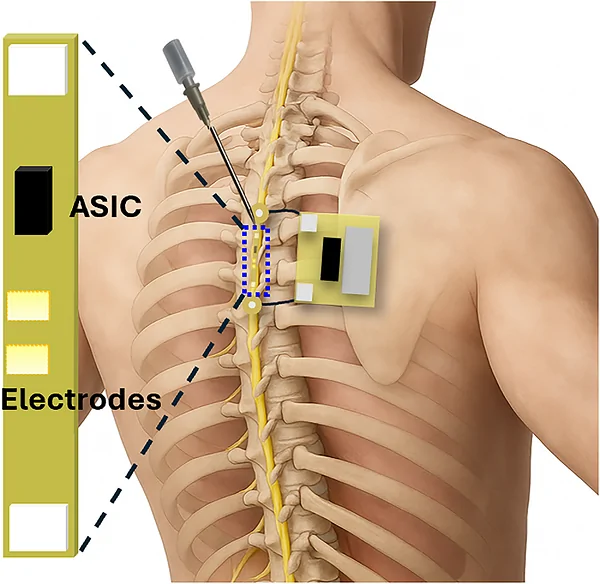

## 1 Introduction

Electroceuticals are gaining traction as therapeutic tools for disorders of the central and peripheral nervous systems. Within the central nervous system (CNS), spinal cord stimulation has demonstrated efficacy in treating chronic pain and movement impairments. In the peripheral nervous system (PNS), vagus, sacral, and tibial nerve stimulation have been used to manage inflammatory, gastrointestinal, and urinary disorders, while somatic nerve interfaces restore motor and sensory function in paralyzed or injured limbs [1]. Collectively, these neural electroceuticals offer precise, reversible, and localized control of physiological functions, providing therapeutic alternatives where conventional pharmaceuticals are ineffective or poorly tolerated [2].

Current spinal or peripheral nerve stimulators require open surgical implantation to accurately position leads on millimeter‐scale nerves, ensuring stable contact and avoiding off‐target activation [3, 4]. Furthermore, these systems are bulky, necessitate invasive procedures and implanted pulse generators (IPGs), and are poorly suited for distributed or multi‐site interfacing. The invasiveness and duration of these surgical procedures often relegate implantable electroceuticals to use only as a last‐line intervention when conventional therapies fail.

As summarized in Figure 1a, existing nerve stimulation strategies can be broadly categorized by their invasiveness, efficiency, and long‐term reliability. (A) Transcutaneous electric nerve stimulation (TENS) provides excellent safety and noninvasiveness but suffers from poor spatial precision and low coupling efficiency through tissue [5]. (B) Injectable wireless stimulators combine wireless powering with minimally invasive delivery, allowing small implants to be placed close to target nerves through a fine needle [6, 7, 8, 9, 10, 11]. (C) Implantable wirelessly powered stimulators eliminate leads but still require pocket formation and precise surgical placement [12, 13, 14, 15, 16, 17, 18, 19]. (D–E) Lead and cuff electrode systems integrate onboard batteries and wired electrodes, delivering the highest efficiency and chronic stability, but at the cost of extensive tunneling and high surgical invasiveness [20, 21, 22]. Surgical tunneling of electrodes and subcutaneous pulse generators introduces heavy clinical burden, while tethered connections limit the placement and number of stimulation sites. As a result, the past decade has witnessed a shift toward wireless power transfer (WPT) technologies for implantable medical devices (IMDs). These approaches enable smaller form factors, reduced device weight, extended operational longevity, and, critically, less invasive implantation, thereby positioning categories (B‐C) as the natural evolutionary directions for future systems.

**FIGURE 1:**
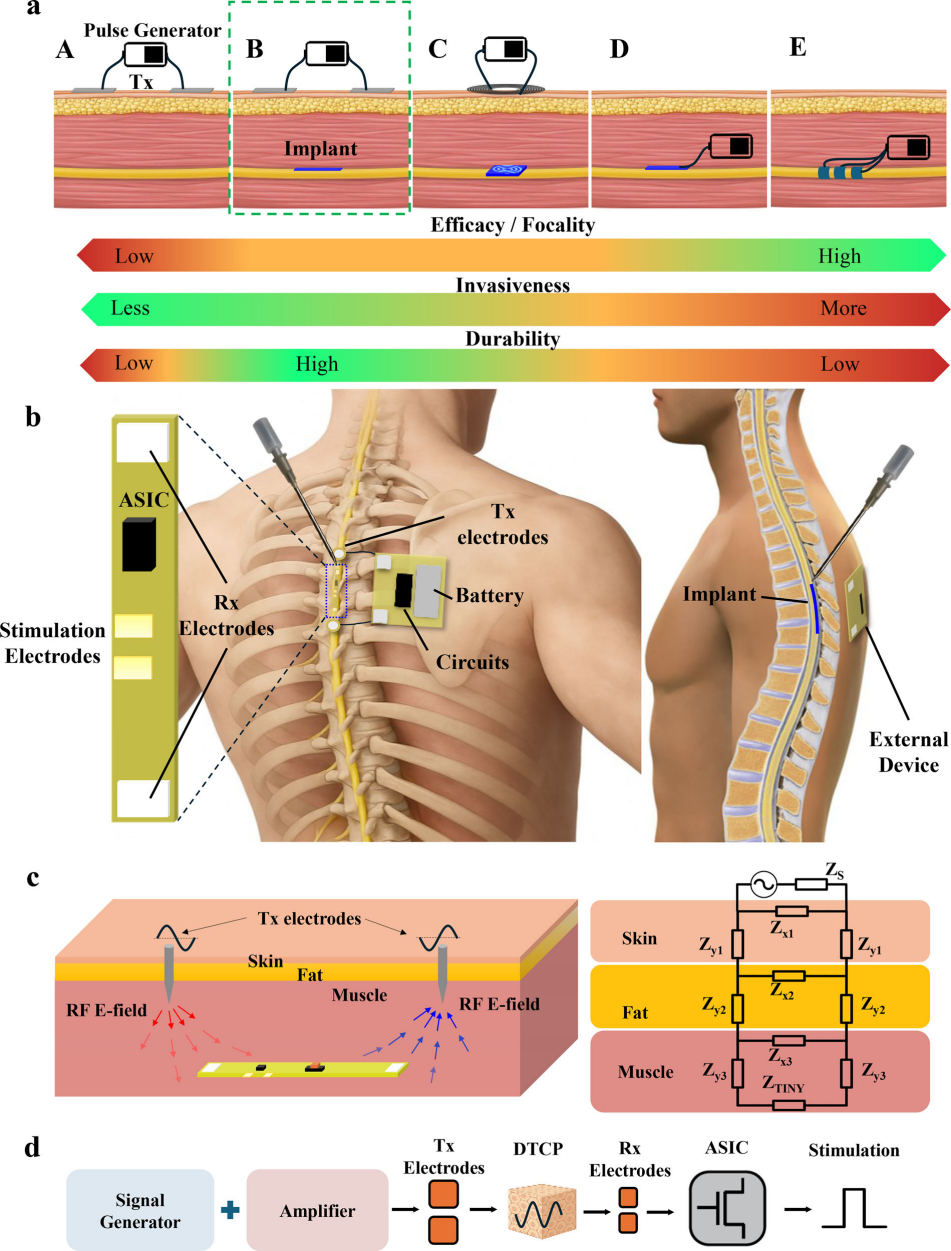
Overview of the DTCP injectable neural stimulator. (a) Comparison of representative nerve‐stimulation interfaces in terms of efficacy/focality, invasiveness, and durability: A. Transcutaneous electric nerve stimulation (TENS); B. Injectable wireless stimulator; C. Implantable wireless stimulator; D. Implanted lead stimulator; E. Implanted cuff electrodes. (b) Concept of a TINY device injected near a representative nerve target and powered by a portable external transmitter through DTCP. TINY device integrates Rx electrodes, stimulation electrodes, and an ASIC. (c) Working principle of DTCP and a simplified conductive–capacitive tissue‐coupling model. External differential Tx electrodes drive an RF‐frequency field, while the Rx electrodes harvest the local differential potential. Tissue‐layer impedances affect coupling efficiency. (d) Block diagram of the overall electronic system, consisting of the external RF generator, tissue coupling path, and the circuits on the implant to convert RF energy to DC stimulation.

However, while WPT represents a major step toward reducing surgical burden, achieving true minimal invasiveness requires not only eliminating leads and batteries but also eliminating open surgical dissection and pocket creation. The emerging concept of the injectable neurostimulator aims to meet this goal by delivering ultra‐miniaturized stimulators directly into target tissues through a fine needle, avoiding open surgery and subcutaneous tunneling altogether. (B) Injectable systems, combining minimally invasive delivery with efficient and localized stimulation, offer the most balanced trade‐off between safety, adaptability, and performance, making them the most promising direction for next‐generation electroceutical systems.

Unfortunately, conventional WPT methods, such as inductive, ultrasonic, magnetoelectric, and optical coupling, face significant challenges in human‐centric injectable scenarios. Magnetoelectric (ME) and optical approaches, while attractive for efficiency or precision, are difficult to reconcile with needle‐deployable form factors. ME systems typically rely on resonant Metglas–PZT laminates whose required thickness and encapsulation substantially increase device volume [23, 24], whereas optical links suffer from severe tissue scattering, limiting penetration to sub‐millimeter depths with insufficient power delivery [25, 26, 27, 28]. Ultrasonic links can power millimeter‐scale stimulators at centimeter depths, benefiting from low acoustic attenuation in soft tissue [6, 7, 8]. However, the need for precise transducer alignment and coupling gel severely limits practical usability. The external acoustic heads remain bulky, and lateral misalignment quickly degrades coupling efficiency. As a result, it remains unsuitable for distributed or fully wearable injectable systems. Meanwhile, RF mid‐field and inductive approaches have been explored to advance miniaturized, battery‐free stimulators. For example, *Neuspera* developed a mid‐field inductively powered sacral nerve stimulator capable of activating deep neural targets in human trials [9, 10], though the device remains too large (50 mm^3^). More recently, Fuglevand et al. presented a smaller ferrite‐core coil stimulator (7 mm^3^) intended for injectable use [11]; however, its rigid structure and strong orientation sensitivity complicate compliant implantation and robust operation. Notably, these studies provide limited quantitative power‐transfer efficiency (PTE) characterization, underscoring the difficulty of achieving both efficient RF coupling and true injectability. Alternatively, *Injectrode* (Neuronoff Inc.) [29, 30] employs a syringe‐delivered conductive path to couple with transcutaneous stimulation, but its millimeter‐scale wire and lack of integrated circuitry hinder scalability and future closed‐loop functionality. Overall, existing approaches remain constrained by inherent trade‐offs between efficiency, depth, robustness, and form factor, motivating an injectable‐oriented tissue‐coupled powering architecture that can better balance efficiency, depth, alignment tolerance, and needle‐compatible form factor.

Our proposed strategy, Differential Tissue‐Coupled Powering (DTCP), is an engineered MHz‐band tissue‐coupled powering architecture based on volume‐conduction principles. In DTCP, external differential electrodes placed near the implant site drive an RF‐frequency electric field/current through tissue, and implanted receiver electrodes harvest the local differential potential to power active electronics. Although the underlying tissue‐field behavior can be modeled by classical volume conduction theory, the term “volume conduction” is commonly used in clinical neurophysiology to describe passive propagation and recording of bioelectric potentials from endogenous sources [31], whereas “galvanic” terminology often emphasizes conductive current flow through tissue [32]. We therefore use “tissue‐coupled powering” to emphasize the complete engineered Tx–tissue–implant conductive–capacitive power‐delivery architecture, including external field generation, differential pickup, rectification, and local stimulation. By separating the MHz‐band power carrier from the locally generated stimulation waveform and operating within established exposure limits, DTCP enables miniaturized, battery‐free injectable devices that can be powered by compact external transmitters while retaining minimally invasive deployment.

Prior volume‐conduction and body‐coupled prototypes have demonstrated neural recording, stimulation, or powering using surface/needle Tx electrodes coupled to implantable receivers [33, 34, 35, 36, 37, 38]. The work represented by [33] demonstrated implantable stimulation using high‐frequency volume‐conduction powering; however, its implant remains ∼16× larger and exhibits ∼8.6× lower PTE than TINY under the reported conditions. The systems represented by [37] demonstrate body‐coupled power delivery and neural recording, but they have not shown stimulation or needle‐based injectable deployment capability, and their receivers remain larger and less efficient than TINY. As summarized in Table S1, TINY is compared with these and other academic systems in terms of operating frequency, implant volume, PTE, depth, injectability, external hardware, and depth/volume FoM. Commercial neuromodulation systems, including StimRouter [39, 40, 41], Curonix/Freedom PNS [42, 43, 44], Nalu Neurostimulation [45, 46], NuStim [47, 48, 49, 50], and Neuspera iSNM [51, 52, 53, 54], provide important clinical benchmarks. However, as summarized in Table S2, these systems generally rely on lead‐based implants, larger receiver modules, or non‐injectable form factors rather than mm^3^‐scale injectable active stimulators.

To address this gap, we develop Thread‐like Injectable Neural TechnologY (TINY), an end‐to‐end DTCP platform that integrates a custom ASIC, power‐receiving electrodes, and stimulation electrodes into a flexible strand for needle‐based deployment near target nerves (Figure 1b). TINY leverages DTCP length scaling to increase harvested power primarily through receiver length while preserving a small cross‐section, and it uses a compact battery‐powered external transmitter to support wearable operation.

Building on this DTCP‐enabled architecture, this work makes four key contributions.

1. Length‐dominant DTCP power scaling and length–depth optimization for injectable receivers: We experimentally validate DTCP length scaling under co‐optimized Tx–Rx geometry, showing that increasing receiver‐electrode spacing improves harvested power/PTE while preserving a needle‐compatible cross‐section. Unlike fixed‐Tx studies where longer Rx spacing can simply move part of the receiver closer to the transmitter, our measurements optimize the Tx–Rx configuration for each receiver length and depth, revealing a practical saturation behavior rather than an unlimited “longer‐is‐better” trend. This establishes a length–depth design rule for selecting receiver length to balance delivered power against injection, placement, and recovery constraints in injectable electroceuticals.
2. Ultra‐miniaturized flexible thread‐like implant: Leveraging this length‐scaling strategy, we demonstrate a flexible, thread‐like injectable stimulator with an implant volume of 2.3 mm^3^ (≈ 11× smaller than prior volume‐conduction‐powered devices), integrating receiver electrodes, stimulation electrodes, and a custom ASIC while maintaining functional neural stimulation capability.
3. Quantitative DTCP design rules and experimentally validated robustness characterization: We characterize key DTCP design parameters including carrier frequency, electrode surface, transmitter electrode spacing, receiver length, implant depth, and receiver‐length saturation. We further experimentally quantify PTE changes under axial rotation and tilt‐induced misalignment, which, to our knowledge, has not been systematically validated in prior tissue‐coupled/volume‐conduction powering studies. These results establish practical guidelines for DTCP optimization and injectable deployment.
4. Portable transmitter and in vivo validation: We implement a compact battery‐powered DTCP transmitter and demonstrate wireless peripheral nerve stimulation in rats, device injection/placement feasibility, 20‐day post‐injection positional assessment, and 5‐day functional survival stimulation with an active device.

## 2 Results

### 2.1 DTCP Coupling Principle and System Concept

DTCP is an engineered tissue‐coupled powering architecture based on volume‐conduction principles, in which external differential electrodes drive an RF‐frequency electric field/current through tissue and implanted electrodes harvest the local differential potential [55].

Figure 1b illustrates the conceptual design of the TINY system (shown here for a representative spinal nerve target as an example application, but applicable to other peripheral targets). Conventional spinal cord stimulation typically relies on surgically implanted lead systems and an implantable pulse generator, requiring tunneled leads and a relatively invasive implantation procedure. In contrast, the TINY takes a different approach: a ∼1.5–2 cm thread‐like implant is injected directly into the desired target region using a customized needle, while a compact, battery‐powered wearable controller placed on the overlying skin provides the excitation. This architecture enables minimally invasive deployment without implanted batteries or tunneled leads, while retaining localized stimulation near the neural target.

The operating principle of DTCP is to establish an alternating E‐field in tissue using two external transmitter (Tx) electrodes and to differentially harvest the resulting potential drop with two receiver (Rx) electrodes on the implant. When a sinusoid in the MHz range is applied across the Tx pair, both conductive and displacement currents contribute to the tissue‐coupled path. While the multilayer tissue structure influences the effective impedances and local field partitioning, the coupling occurs in a quasi‐static conductive–capacitive regime rather than through far‐field radiation. This tissue‐mediated coupling is further supported by the ex vivo pork‐tissue validation in Figure S1, where power transfer was observed through tissue but not under the no‐tissue condition. Thus, “RF” here refers to the carrier frequency and circuit operation, not to a radiative antenna‐based power link. The implant harvests the local field and converts the differential voltage into useful power.

To interpret coupling and guide design, the tissue volume between the two electrode pairs can be approximated by a layered lumped network (right panel in Figure 1c). Each effective layer—skin, fat, and muscle—is represented by a complex vertical impedance (*Z*_y1_, *Z*_y2_,*Z*_y3_) and a transverse impedance (*Z*_x1_,*Z*_x2_,*Z*_x3_), capturing both conductive and capacitive components in the tissue‐coupled path. Consequently, the delivered power at the implant depends on (i) the Tx spacing *d*_Tx_ relative to Rx spacing *d*_Rx_, (ii) implant depth and local layer thicknesses, and (iii) frequency, which together determine the local field gradient and the differential voltage available across the Rx electrodes.

This model provides a design framework for interpreting two experimentally observed features of DTCP. First, DTCP exhibits length‐dependent harvesting: increasing *d*_Rx_ generally increases PTE, even when *d*_Tx_ is increased proportionally. As *d*_Rx_ grows, the vertical impedance terms remain primarily governed by implant depth and local tissue layers, whereas the transverse impedance terms increase with lateral separation. Thus, a longer receiver pair samples a larger fraction of the local potential gradient and increases the harvested power. However, this benefit gradually saturates with *d*_Rx_, so further length extension must be balanced against injection, placement, and recovery constraints. Second, DTCP is tolerant to angular misalignment because the implant samples the potential difference between two points in tissue, avoiding direct dependence on coil orientation or an acoustic focal spot.

In addition to the coupling model, Figure 1d summarizes the end‐to‐end system signal and power flow of the DTCP–TINY system. A sinusoidal excitation generated by the RF source and amplified by the external driver is applied across the Tx electrodes, establishing a differential electric field in tissue. The implant samples this field with its Rx electrodes and converts the differential RF voltage to a rectified supply through an on‐chip RF‐to‐DC front end, which is then regulated/limited to provide a stable supply for the stimulation circuitry. In this way, the block‐level view links the tissue‐coupling mechanism in Figure 1c to the implant implementation described in Section 2.2 and the PTE/robustness results quantified in Section 2.3.

### 2.2 TINY Implementation and Injectable Form Factor

To evaluate the powering performance of the TINY implant, we establish a battery‐isolated benchtop setup using differential RF excitation and real‐time power monitoring (Figure 2a). The entire system is powered by a floating battery supply to minimize ground‐coupling artifacts, and the single‐ended RF signal from the generator is amplified and then split by a 180° –3 dB hybrid coupler into differential outputs. All subsequent connections use the same type and length of coaxial cables to ensure balanced transmission. Bidirectional couplers are inserted to measure forward and reflected power in real time.

**FIGURE 2:**
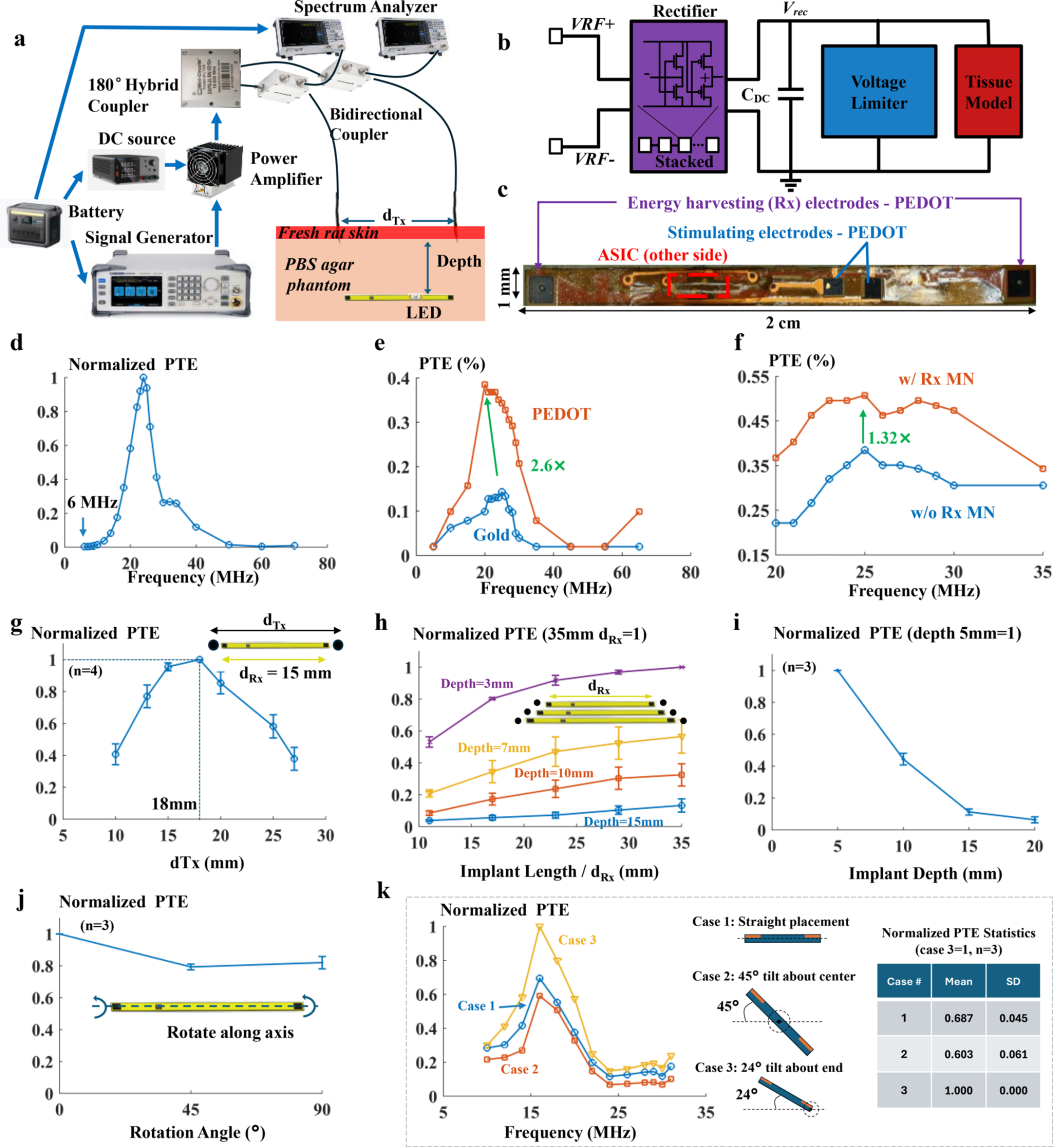
Benchtop characterization of TINY (a) Schematic of the bench test setup. A 180° –3 dB hybrid coupler converts the single‐ended signal into a differential output, followed by equal‐length coaxial cables for balanced transmission. Same bidirectional couplers monitor forward and reflected power in real time, enabling accurate estimation of the PTE. The phantom consists of a fresh rat skin placed over PBS agar. (b) ASIC block diagram showing the impedance matching network, stacked cross‐coupled rectifier, and voltage limiter for overvoltage protection. (c) Photo of the implant with ASIC and PEDOT‐coated energy‐harvesting and stimulation electrodes. The device has a total volume of 2.3 mm^3^ and a weight of 8 mg. The LED is mounted on the stimulation electrodes for bench test only. (d) PTE–frequency sweep showing the MHz‐band operating window. The LED could not be powered below 6 MHz in the setup of panel a, while higher PTE was observed in the 16–28 MHz range. (e) PTE–frequency comparison of gold and PEDOT‐coated surfaces. The PEDOT coating provides 2.6 × efficiency. (f) Implant with matching network (MN) shows 32% higher efficiency than the one without. (g) Optimal spacing between the Tx electrodes (dTx) for an implant with dRx = 15 mm at an implantation depth of 10 mm. PTE was normalized to the maximum value at dTx = 18 mm within each independently fabricated device. Data are shown as mean ± SD from four devices. (h) Normalized PTE as a function of implant length/dRx at different implantation depths. For each dRx and depth, the Tx–Rx relative position was optimized, and the frequency was swept to extract the maximum PTE. The PTE increase gradually saturates at longer dRx, indicating diminishing benefits from further length extension. Repeatability was evaluated using two independently fabricated devices and two independently prepared phantom, with individual normalized traces shown in Figure S7. (i) PTE versus implant depth in the phantom, normalized to the 5 mm‐depth value within each independently fabricated device. Data are shown as mean ± SD from three independently fabricated devices. (j) PTE under rotation along the implant axis from 0° to 90°, normalized to the 0° case within each independently fabricated device. Data are shown as mean ± SD from three devices. The maximum reduction in normalized PTE is 20.7%, indicating tolerance to axial rotation. (k) PTE under tilted implantation conditions. PTE was normalized to Case 3 within each independently fabricated device before calculating the mean and SD across devices. Case 1 and Case 2 show comparable normalized PTE values, with Case 1 approximately 14% higher than Case 2. Data are shown as mean ± SD from three independently fabricated devices. For panels g, i, j, and k, repeated sweeps were first averaged within each device, and the device‐level normalized values were used to calculate the mean and SD across independently fabricated devices. Panel g was measured using four devices, and panels i, j, and k were measured using three devices. For panel h, repeatability was evaluated using two independently fabricated devices and two independently prepared phantoms, giving three individual normalized traces.

Wireless power reception is benchmarked using an integrated LED indicator as a repeatable threshold condition. PTE is defined as the ratio of the received power at this threshold to the net RF power injected at the Tx electrode port (see Experimental Section for details).

Bench measurements are conducted in controlled tissue‐mimicking phantoms. Unless otherwise specified, the phantom consists of 1× PBS diluted 20‐fold with 1.8% agar, providing stable and reproducible coupling conditions with an effective conductivity close to that of muscle (∼0.5 S/m) [56]. For the updated length/depth characterization in Figure 2d,h, a fresh rat‐skin layer was placed over the PBS‐agar phantom to better approximate the skin/tissue interface. The PBS‐agar phantom is used primarily for repeatable parameter sweeps and design optimization (e.g., frequency, *d*_Tx_, *d*_Rx_, depth, and misalignment), whereas fresh‐skin and ex vivo tissue measurements provide supplementary validation under more tissue‐relevant conditions.

The custom‐fabricated ASIC (Figure 2b) integrates an RF‐to‐DC rectifier and an over‐voltage protection circuit. We employ a four‐stage cross‐coupled differential‐drive (CCDD) rectifier to achieve efficient RF energy harvesting while presenting an appropriate input impedance under the stimulation load. A crowbar‐style voltage limiter protects the downstream circuits by rapidly sinking current once the rectified supply exceeds a preset threshold, while remaining inactive during normal operation to minimize standby loss (Figure S2). Since stimulation represents the highest power‐demand operating mode, the rectifier is sized and optimized for peak efficiency under this condition. The diced die footprint is 0.6 × 0.3 mm^2^.

The implant (Figure 2c) consists of the ASIC and PEDOT‐coated energy‐harvesting and stimulation electrodes. The device is 2 cm long and 1 mm wide on a 0.1 mm‐thick flexible PCB, with a total volume of 2.3 mm^3^ and a mass of 8 mg. Because an optical indicator is unnecessary in vivo (light is not visible through tissue), the LED is omitted from the implant layout. Instead, for benchtop characterization, an LED is temporarily mounted across the stimulation electrodes to provide a visual readout of harvested power and stimulation activity.

### 2.3 Benchtop Characterization and DTCP Design Rules

We first characterize the frequency dependence of DTCP power transfer using the bench setup in Figure 2a. As shown in Figure 2d, LED‐threshold activation was not achieved below 6 MHz under the available drive condition, whereas the PTE increased markedly in the MHz range and showed a favorable window around 16–28 MHz. The exact optimum varies with tissue condition, electrode contact, Tx/Rx geometry, and implant loading. We therefore use 25 MHz as a representative operating frequency for subsequent characterization, while noting that the optimum is system‐ and tissue‐condition‐dependent rather than a fixed universal value. To further rationalize this favorable MHz operating window beyond the empirical PTE sweep, we developed a simplified impedance‐based DTCP circuit model (Figure S3). This lumped model is not intended to reproduce the exact PTE spectrum, but it shows that the low‐frequency regime is limited by the large effective tissue‐coupled impedance of the electrode–tissue pathway, while the estimated load power increases in the MHz range and then becomes bounded by the non‐monotonic impedance response, tissue‐path loss, parasitic coupling, and implant loading. This behavior supports a bounded, system‐dependent MHz operating window rather than a universal single optimum frequency.

This MHz‐band choice also avoids using kHz/sub‐MHz currents as the wireless power carrier, since stimulation and conduction‐block studies have shown that currents in these frequency ranges can directly modulate neural excitability [57, 58, 59]. In DTCP, the carrier is used only for power delivery, while the implant locally generates the controlled stimulation waveform. This separation enables programmable stimulation parameters and leaves room for future functions such as recording, sensing, closed‐loop control, or multi‐node addressing without requiring the external power waveform itself to serve as the neuromodulatory signal.

To lower the Rx electrode impedance and improve the electrode–tissue contact, we electroplate PEDOT onto the gold electrodes. As shown in Figure 2e, PEDOT coating increases PTE within the optimal frequency range by 2.6× relative to the uncoated electrodes, with noticeable gains across the entire measured band.

To further improve PTE, an impedance matching network (MN) is incorporated into the DTCP receiver path. An L‐type configuration with minimal components is implemented to balance performance and implant size. As shown in Figure 2f, the MN improves the PTE at 25 MHz from 0.38% to 0.51% (a 32% relative improvement) for a 15 mm implant at 10 mm depth, compared to the unmatched case. Although ideal matching suggests much higher enhancement, the limited Q‐factor (< 5) of the required high‐value inductors (∼5 µH) introduced considerable loss, constraining the achievable gain. Moreover, the discrete components are larger in size than the ASIC, offsetting the benefits of miniaturization. These results indicate that, under the current design constraints, impedance matching provides measurable but modest improvement; however, its impact is expected to increase as system capacitance is further optimized in future miniaturized implementations.

We next analyze geometric parameters affecting PTE. For an implant with Rx electrode spacing (dRx) of 15 mm placed at a depth of 10 mm (Figure 2g), measurements from four independently fabricated devices showed an optimal Tx electrode spacing (dTx) between 15 and 20 mm, with the maximum normalized PTE occurring at dTx = 18 mm. The device‐to‐device variation after normalization was limited, and all devices preserved the same optimal spacing trend. The individually normalized curves from the four devices are provided in Figure S4. This reproducible trend is consistent with the field‐distribution interpretation in Section 2.1. The largest potential change occurs primarily in the region between the two Tx electrodes, whereas the potential varies more slowly in the immediate vicinity of each electrode (where it is effectively clamped by the electrode boundary condition). The Rx pair harvests power from the differential voltage across its electrodes, which corresponds to the line integral of the local electric field over the receiver spacing. If *d_Tx_* = *d_Rx_* , the ends of the Rx pair are more likely to fall into near‐electrode regions where the potential gradient is reduced, so a portion of *d_Rx_* contributes less to the harvested voltage. Choosing *d_Tx_* slightly larger than *d_Rx_* positions the Rx pair predominantly within the high‐gradient region between the Tx electrodes, maximizing the sampled differential voltage and improving PTE. HFSS simulations of the E‐field distribution further support this interpretation (Figure S5). When *d*_Tx_ becomes excessively large, tissue losses increase, and the Rx electrodes can no longer effectively harvest the full potential difference along the dominant field path. Therefore, for this implant geometry and depth, the optimal *d_Tx_* is typically slightly greater than *d_Rx_*.

Building on the DTCP coupling analysis above, we next examine how power transfer depends on receiver length under different depths. For each *d_Rx_* and implantation depth, the Tx–Rx relative position was optimized, and the frequency was swept to extract the maximum PTE. As shown in Figure 2h, increasing *d_Rx_* improves normalized PTE at all tested depths, confirming that a longer receiver samples a larger differential tissue potential. However, the PTE increase gradually saturates at longer *d_Rx_* at all depths, indicating that receiver length is not an unlimited design variable. This length‐dependent trend was also confirmed by three individual normalized traces obtained from two independently fabricated devices and two independently prepared phantoms using the same recipe (Figure S6), supporting that the observed saturation behavior is not specific to a single implant or phantom preparation. This saturation is important for injectable systems because further length extension increases injection‐tool requirements, placement difficulty, and potential post‐implantation mechanical burden while providing diminishing power gains. Therefore, the experimentally validated DTCP length scaling provides a length–depth design rule for injectable receivers, rather than simply indicating that implant length should be maximized. Additional receiver‐length characterization under optimized Tx–Rx alignment is provided in Figure S7, where both HFSS simulation and measurement show a consistent monotonic PTE increase with increasing *d_Rx_* at 15 mm depth.

Importantly, this length‐scaling strategy does not require a proportional increase in implant cross‐sectional area: the implant width can remain nearly unchanged as *d_Rx_* is extended. Although the commercial flexible PCB used here is not precision‐trimmed due to vendor fabrication limits, an in‐house substrate platform [60] could extend receiver length with negligible increase in cross‐section and minimal volume penalty. Thus, DTCP provides a distinctive route to increasing harvested power primarily through implant length, while preserving the needle‐compatible cross‐section required for injectable deployment.

Depth effects are also quantified. Figure 2i shows the depth dependence of DTCP power transfer for a 15 mm implant with PEDOT coating and MN optimization, where PTE is normalized to the 5 mm‐depth condition (1.25% at 25 MHz, set to 1). Measurements from three independently fabricated devices show a consistent decrease in normalized PTE with increasing implantation depth. The individually normalized curves from the three devices are provided in Figure S8. We note that this trend is reported for a fixed 15 mm implant; longer implants provide a higher baseline coupling due to DTCP length scaling and can partially offset depth‐induced attenuation. Moreover, even at 20 mm depth, the received power can be increased by raising the injected Tx power, since the measured contact current remains well below applicable safety limits (Section 2.4).

Another major advantage of DTCP is its tolerance to implant orientation. As shown in Figure 2j, rotating the implant around its longitudinal axis from 0° to 90° reduces the normalized PTE by only 21% (from 1 to 0.79). Measurements from three independently fabricated devices show the same rotation‐tolerant trend, with the individual normalized curves provided in Figure S9. This robustness arises because DTCP harvesting depends primarily on the potential difference between two receiver contact points in tissue; axial rotation largely preserves the spacing and depth of these contacts relative to the surrounding field, resulting in minimal efficiency change. In contrast, coil‐based links are strongly orientation‐dependent: rotations that misalign the coil normal with the magnetic field can substantially reduce coupling efficiency. These results highlight the strong tolerance of DTCP to implant rotation.

We further evaluate tilt‐induced misalignment about the implant. Three representative cases are tested in Figure 2k: Case 1 is the straight, flat placement at 10 mm depth; Case 2 tilts the implant by 45° about its midpoint; and Case 3 tilts the implant by 24° about the right end. Case 3 shows the highest PTE because one side of the implant moves closer to the Tx electrodes, increasing the local field pickup. Therefore, the peak PTE in each device was normalized to its Case 3 value for cross‐device comparison. Because the peak frequency varied slightly among independently fabricated devices, Figure 2k shows a representative frequency sweep from one device, together with the mean and SD of the peak normalized PTE values extracted from three independently fabricated devices. For comparison, an ideal coil‐based inductive link would exhibit an orientation‐dependent power transfer approximately proportional to 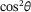, corresponding to a 50% power reduction at 45° misalignment. In contrast, Case 2 shows only a 12.2% reduction compared with the straight Case 1, indicating that DTCP is relatively tolerant to tilt misalignment. The individual frequency sweeps from the three devices are provided in Figure S10, with each device showing the same relative trend among the three tilt cases. This tolerance arises because the receiver harvests the local differential tissue potential rather than relying on coil‐axis alignment or antenna orientation.

### 2.4 Portable Transmitter and Safety‐Relevant Characterization

To move toward a wearable system for untethered neural stimulation, we developed a battery‐powered transmitter prototype. Figure 3a outlines the architecture: an MCU (MSPM0C1104) programs a direct digital synthesis (DDS) chip (AD9850) to generate a low‐power RF‐frequency carrier with MCU‐controlled frequency and amplitude (the latter also adjustable via an onboard potentiometer). The battery output is boosted by an LTC3122 converter to supply an RF power amplifier (PMA3‐43‐1 W) that provides approximately 22 dB of gain. The amplified signal then passes through a balun (MABAES0060) to generate a differential output driving two Tx electrodes. As shown in Figure 3b, the fabricated prototype measures 63 mm × 38 mm and is powered by a flexible 3.7 V lithium battery. Applying a pulsed drive to the power amplifier (500 µs/s) yields an average system power consumption of 0.039 W, corresponding to an estimated operating time of ∼17 h with a 200 mAh ultrathin flexible battery. A wireless charging block can be integrated in future versions to extend operating time. Both pulse width and stimulation frequency are fully programmable via the MCU for flexible protocol control. The system attains a maximum output power of 28 dBm before Tx electrodes (Figure 3c; measured with a 20 dB attenuator on the spectrum analyzer). Although fabricated on FR‐4, the same design can be migrated to a flexible PCB to enhance wearability.

**FIGURE 3:**
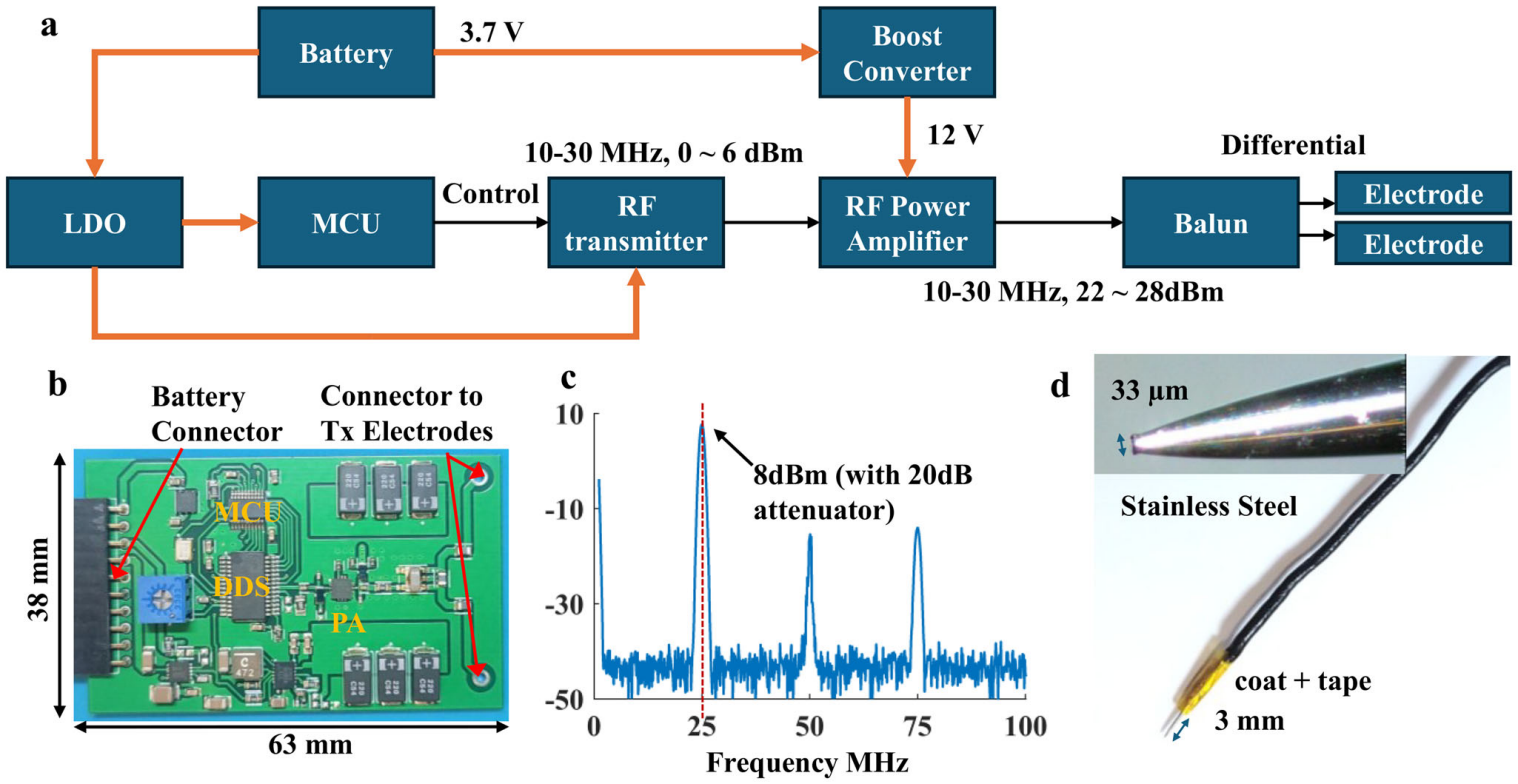
Portable DTCP transmitter system for wireless powering of injectable implants. (a) System block diagram of the battery‐powered wireless transmitter. The circuit integrates an MCU‐controlled DDS to generate a programmable RF carrier, followed by a boost converter and RF power amplifier to drive a balun‐connected differential output for the external Tx electrodes. (b) Photograph of the fabricated prototype (63 mm × 38 mm) that operates from a 3.7 V lithium battery and supports programmable pulse‐modulated operation. (c) Measured RF output spectrum demonstrating a maximum transmitted power of 28 dBm at fundamental frequency, using 25 MHz as an example (with a 20 dB attenuator). (d) Micro‐pin Tx electrode designed to enhance tissue coupling with minimal invasiveness. The stainless‐steel electrode features a 33 µm tip width, 3 mm exposed length, and 330 µm maximum diameter at the base end—smaller than typical CGM needles—coated with PDMS and wrapped with polyimide for insulation and robustness.

The Tx electrodes, shown in Figure 3d, are designed to improve PTE while minimizing invasiveness. Because the conductivity of skin is low [56], substantial coupling loss occurs through these layers. To address this, a micro‐pin stainless‐steel electrode is developed to penetrate the skin and enhance coupling efficiency with minimal tissue disruption. The pin features a 33 µm tip width, 3 mm exposed length, and 330 µm maximum diameter, smaller than commercial continuous glucose‐monitoring (CGM) needles, supporting its minimally invasive profile. The proximal end of the pin is press‐fit into a copper lead and soldered at the junction. The remaining exposed portion is coated with PDMS and wrapped with polyimide tape to provide waterproofing and maintain mechanical robustness during implantation. Additional benchtop measurements using non‐invasive surface electrodes are presented in Figure S1. While surface coupling is feasible, stable fixation on rat skin proves challenging and limits repeatability; therefore, pin electrodes are adopted in the main experiments to ensure repeatable conditions.

At the representative 25 MHz operating condition, the IEEE C95.1 contact‐current limit is 31.5 mA [61]. The electrode–tissue impedance measured on a rat hindlimb using a VNA ranges from 325 Ω to 362 Ω in resistance and from 27.2 pF to 38.6 pF in capacitance. These values vary due to differences in electrode–tissue contact and micromotion from respiration. With 28 dBm of RF power, considering a stimulation duty cycle of 500 µs/s, the resulting RMS current is only 0.61 mA, substantially below the corresponding safety limit. A detailed derivation of the corresponding current and power levels is provided in Note S1. In addition, tissue safety simulation of specific absorption rate (SAR) yields 0.82 W/kg for continuous‐wave (CW) transmission at 1 W, remaining below the applicable safety limit (Figure S11).

### 2.5 In Vivo Peripheral Nerve Stimulation and Short‐Term Implantation Validation

To evaluate in vivo functionality and implantation feasibility, we performed complementary animal studies with the TINY system (Figure 4): acute wireless sciatic nerve stimulation, device injection/placement validation, 20‐day post‐injection positional assessment, and short‐term survival stimulation with an active device. Together, these experiments evaluate implant‐mediated peripheral nerve activation, injectable placement feasibility, short‐term positional stability, and functional stimulation persistence over several days.

**FIGURE 4:**
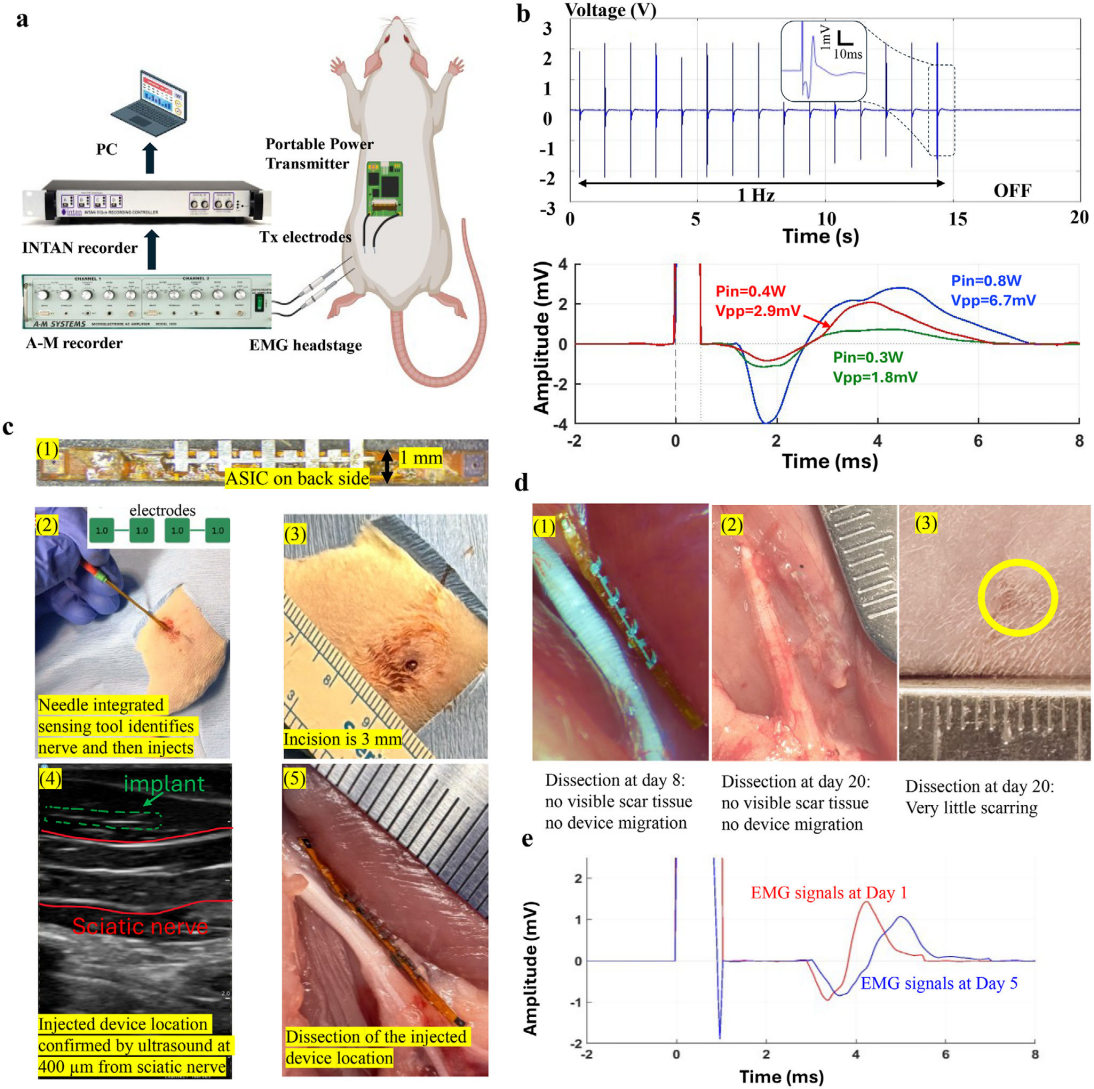
In vivo demonstration of wireless peripheral nerve stimulation and post‐injection stability of the TINY system. (a) Experimental setup for wireless sciatic nerve stimulation in rats using the portable DTCP transmitter. The transmitter drives two external Tx electrodes while EMG signals are recorded from the biceps femoris muscle. The implant is positioned adjacent to the sciatic nerve. (b) Wireless stimulation evokes repeatable EMG signals at 1 Hz. Representative EMG trace shows time‐locked muscle activation synchronized with each stimulus pulse (500 µs), and EMG amplitudes increase with higher net RF input power. The reported power levels of 0.3–0.8 W were calculated as forward power minus reflected power, measured at the Tx electrode input. The Tx–Rx separation was approximately 10 mm, and the powering frequency was 20 MHz. (c) Injection and placement validation using the TINY implant. (1) Optical micrograph of the 1 mm device with biological anchors to reduce post‐injection migration (2) Integration of the nerve sensor into a 14‐gauge spinal needle for injection. (3) Photograph of a 3 mm skin incision prior to injection. (4) Ultrasound imaging confirming device placement (green) adjacent to the sciatic nerve (red). (5) Dissection image confirming the injected device location. (d) Post‐injection tissue images from the 20‐day dummy‐device study. (1) 8 days after injection, the implant remains at the intended location without migration or encapsulation. (2) At 20 days, the implant continues to reside near the nerve with no visible fibrotic reaction. (3) External skin inspection at day 20 shows only a small residual mark (yellow circle), indicating minimal invasiveness. (e) Short‐term survival functional stimulation study using an implanted active device. EMG responses recorded on day 1 and day 5 show successful wireless stimulation after implantation, demonstrating short‐term functional stability under the tested conditions.

Acute wireless stimulation was first tested in rats with the implant positioned adjacent to the sciatic nerve and powered by the portable DTCP transmitter (Figure 4a). The transmitter drove the external Tx electrodes at a representative MHz‐band carrier condition optimized per animal, and EMG signals were recorded from the biceps femoris muscle. A 500‐µs monophasic stimulation pulse train at 1 Hz evoked time‐locked EMG responses and visible hindlimb movement. Increasing the Tx input power increased the stimulation output amplitude and EMG response, supporting the RF‐power dependence of implant‐mediated neural activation (Figure 4b). To exclude direct stimulation by the external field, a negative‐control condition was performed with the same Tx waveform and RF power but with the implant removed; only stimulus‐locked artifacts were observed, with no corresponding EMG (Figure S12). Across acute experiments, wireless stimulation was successfully observed in six rats (*n* = 6). The stimulation capability and the dependence on electrode geometry are further analyzed through finite‐element modelling, as detailed in Figure S13.

Under the representative in vivo stimulation conditions, the maximum available RF power at the rectifier input was estimated to be 29.8 mW, yielding 7.3 mW of rectified DC output power. Based on an implant volume of 2.3 mm^3^, this corresponds to a volumetric power density of 3.17 mW/mm^3^. Using the measured electrode/tissue impedance (Figure S14) and a 3.3 V designed compliance voltage, circuit simulation estimated a peak stimulation current of 2.2 mA.

We next evaluated post‐injection implant placement and migration in rats to assess the positional stability of TINY. This experiment used the TINY implant incorporating the ASIC, PEDOT‐coated electrodes, and a laser‐cut stainless‐steel anchor with a thickness of 50 µm (Figure 4c‐1). These surface features are designed to promote localized tissue anchoring and reduce post‐injection migration. In this placement study, the implant was delivered parallel to the sciatic nerve branches using a nerve‐sensing probe integrated into a 14‐gauge spinal needle through a small skin incision (Figure 4c‐2,3). After the spinal needle was positioned near the nerve, it was advanced deeper into the tissue and aligned parallel to the nerve with assistance from the nerve‐sensing probe. In Figure 4c‐2, the four green pads on the probe form two pairs of sensing electrodes used to detect nerve proximity; the value “1.0” within the pads indicates the model‐predicted probability of nerve tissue after deep‐learning‐assisted signal processing. Saline hydrodissection was performed to create a pocket adjacent to the nerve for implant placement. The implant was then delivered into the pocket parallel to the nerve using a push rod or alginate hydrogel. After implant delivery, the needle was carefully withdrawn while 1 mL of hydrogel was injected along the needle trajectory. Ultrasound imaging and post‐placement dissection confirmed successful placement of the TINY implant adjacent to the sciatic nerve (Figure 4c‐4,5).

Tissue dissection conducted at 8‐ and 20‐days post‐injection confirmed the precise localization of the implanted device, with no observable signs of migration, as shown in Figure 4c. On day 8 (Figure 4d‐1), the device remains securely positioned at the targeted implantation site, with no visible displacement or tissue encapsulation. By day 20 (Figure 4d‐2), the implant continues to maintain its position adjacent to the nerve, further supporting positional stability over the 20‐day observation period. Notably, no apparent fibrotic encapsulation is observed at either time point. In addition, histological assessment (H&E) of the target nerve region on day 20 shows preserved nerve boundary morphology without overt fibrotic capsule formation or neutrophil‐rich inflammatory infiltrate at the epineurium (Figure S15). Additionally, examination of the external skin at the injection site on day 20 (Figure 4d‐3) reveals only minimal surface scarring, suggesting that the implantation method was minimally invasive and well tolerated over the observation period. These results collectively validate the stable in vivo positioning of the device and its tissue compatibility over a 20‐day period.

To further evaluate functional persistence after implantation, we performed a 5‐day survival stimulation experiment using an active TINY device (Figure 4e). In this study, the device was implanted adjacent to the sciatic nerve and wirelessly powered by the external transmitter. EMG recordings showed clear stimulation‐evoked responses on both day 1 and day 5 after implantation, indicating that the implanted device remained functional and capable of peripheral nerve activation over the 5‐day observation period. All surgical procedures are approved by the Institutional Animal Care and Use Committees (IACUC 202500000323) of the University of Florida and Harvard University.

To further assess device‐level stability, we performed accelerated aging tests by immersing three independently fabricated implants in saline at 87°C [62]. The implants retained wireless power reception after accelerated aging, with the longest test reaching a 224‐day‐equivalent aging duration and two additional devices tested to 128‐day‐equivalent and 96‐day‐equivalent durations, respectively; detailed PTE changes are summarized in Figure S16. Even using the largest observed PTE reduction of approximately 22%, restoring the original received power would require only an approximately 1.28‐fold increase in Tx input power, which remains below the measured safety limit discussed in Section 2.4 and Note S1. Therefore, the observed aging‐related PTE loss reduces the received‐power margin but does not remove the available stimulation margin under the tested operating conditions. These accelerated saline tests support short‐term/subacute device‐level stability, while longer‐term chronic implantation will require dedicated packaging optimization and in vivo validation.

## 3 Discussion

This work presents an injectable neural stimulator “TINY” based on DTCP, which is wirelessly driven by a compact, wearable transmitter. TINY integrates the receiver electrodes, ASIC, and stimulation electrodes into a flexible strand deliverable through a fine needle, enabling minimally invasive electroceuticals. Leveraging the differential tissue‐coupled architecture of DTCP, TINY exploits length‐dependent power harvesting, allowing harvested power to increase primarily with receiver length while preserving a needle‐compatible cross‐section, substantially reducing the cross‐sectional penalty typically associated with increasing harvested power in applications requiring higher output. TINY also exhibits strong tolerance to angular misalignment between the external and implanted electrodes. Considering that the implant may shift or rotate within tissue, the external electrodes can be repositioned noninvasively to improve coupling, whereas coil‐ or acoustic‐based links may be more sensitive to implant orientation or focal alignment. Combined with the battery‐powered portable transmitter, these features suggest a path toward wearable and minimally invasive electroceutical systems. A detailed comparison with representative academic wireless‐powering systems and commercial neuromodulation technologies is provided in Tables S1 and S2, showing that DTCP is not intended to maximize PTE alone, but to address the combined requirements of injectable form factor, alignment robustness, and portable external powering.

For a proof‐of‐concept demonstration, TINY is implanted in the rat hindlimb, where wireless stimulation evoked EMG responses and visible leg movements. The portable, battery‐powered transmitter delivers approximately 630 mW power to the external Tx electrodes, and the resulting RMS contact current is 0.61 mA, which remains well below the IEEE [61] and ICNIRP [63] safety limits. Even during transmission bursts, the instantaneous peak current stays within the permissible range. This power budget may support future integration of additional functionalities such as neural recording or biochemical sensing. In addition to improving coupling for small electrodes, MHz‐band operation keeps the wireless power carrier above frequency ranges commonly used for direct neural stimulation or kHz‐frequency conduction block, reducing the likelihood that the carrier itself acts as the neuromodulatory waveform. These results demonstrate wireless, injectable sciatic nerve stimulation in vivo. Further engineering, safety, and chronic functional validation will be required before translation toward biomedical use.

First, to achieve high transmission efficiency in the current setup, we employ a micro‐pin type Tx electrode similar in concept to those used in CGMs. Although the dimension is smaller, this configuration remains invasive. At the human scale, however, optimized Rx electrode designs—such as 3D structures or improved surface coatings to enhance tissue contact—together with better impedance matching on both Tx and Rx sides can collectively raise the overall PTE. With sufficient PTE, effective neural stimulation can be achieved using surface‐mounted external Tx electrodes, allowing the transmitter system to operate in a fully non‐invasive manner.

Second, the present prototype still has significant potential for further miniaturization, as a large portion of the substrate does not contribute to PTE due to the precision constraints of commercial PCB fabrication. Because DTCP benefits from receiver length rather than cross‐sectional enlargement, future microfabrication can further reduce width and thickness while preserving power‐harvesting capability through elongated receiver electrodes.

Third, longer‐term material and functional validation will be needed before chronic implantation. The current prototype uses copper traces encapsulated by polyimide and gold, and potential copper‐ion leakage over prolonged implantation remains to be evaluated. Future in‐house fabrication using Pt or other inert metals could mitigate this concern. Extended active‐implantation studies will also be required to track functional output, encapsulation, and local tissue response over longer time scales.

Beyond the sciatic nerve proof‐of‐concept demonstrated here, DTCP may provide a general powering strategy for other injectable electroceutical platforms, although each target will require dedicated anatomical, safety, and functional validation. For deeper peripheral targets, and potentially for spinal or CNS applications in future studies, the achievable stimulation margin will depend on transmitter‐electrode geometry, safety‐limited RF drive power, tissue thickness, receiver length, rectifier/matching efficiency, and the available on‐implant energy storage. In particular, larger or more efficient on‐implant buffering capacitors could accumulate harvested energy between stimulation pulses and support higher instantaneous stimulation currents, provided that the recharge time and duty cycle remain compatible with the therapeutic protocol. The same tissue‐coupled field could also be explored for distributed or multi‐node injectable implants, where multiple receivers sample the external field at different anatomical locations. Such systems would require node‐specific addressing and communication, for example through frequency‐selective front ends, time‐division powering/stimulation, load‐modulation/backscatter identification, or local control logic. In addition, combined stimulation and recording or sensing systems are possible but would require careful management of RF interference, stimulation artifacts, low‐noise recording windows, and implant power budget. Therefore, while the present work validates DTCP‐powered sciatic nerve stimulation, extensions to deeper peripheral, spinal/CNS, multi‐node, or closed‐loop systems remain future directions that require dedicated system design and in vivo validation.

In summary, DTCP and the TINY system provide a tissue‐coupled powering strategy for battery‐free injectable electroceuticals. By using tissue as a conductive–capacitive pathway for RF‐frequency power delivery, DTCP avoids reliance on tightly coupled coils or acoustic focal paths, which can constrain alignment tolerance and miniaturization. The present work demonstrates this strategy for sciatic nerve stimulation, while future studies could extend the platform toward spinal, dorsal root, vagus, sacral, tibial, or distributed sensor–stimulator interfaces with target‐specific anatomical, safety, and functional validation. Together, these results suggest a route toward minimally invasive, flexible, and wearable‐compatible electroceutical systems.

## 4 Experimental Section

### 4.1 Wireless PTE Characterization

After correcting for the insertion loss of the bidirectional couplers and coaxial cables, the net RF power delivered to the phantom is calculated as *P_in_* = *P_fwd_*  − *P_refl_* where *P_fwd_* and *P_refl_* are the forward and reflected powers referenced to the Tx electrode port. Direct wired measurement of the implant's harvested power is avoided because external leads would extend beyond the implant, perturb the local electric field, and introduce measurement artifacts. Therefore, benchtop characterization employed an LED‐based threshold method to estimate received power without external leads.

To calibrate the threshold, the implant was driven directly at its Rx input while *S*_11_was monitored, and the injected RF power was increased until the LED became just visibly illuminated under constant low‐ambient lighting. The corresponding net RF power delivered to the implant Rx port at this onset condition was defined as the received‐power threshold, *P*_RX, th_ =  343 µ*W*. During wireless experiments, the onset of visible illumination provides a repeatable threshold condition, and the power‐transfer efficiency was computed as PTE  = *P*_RX, th_ /*P*_in_. For the repeatability analysis in Figure 2g–k, repeated frequency sweeps were first averaged within each device to obtain a device‐level response. The device‐level PTE values were then normalized to the corresponding reference condition within each device, and the normalized values were used to calculate the mean and SD across independently fabricated devices. Figure 2g was measured using four independently fabricated devices, while Figure 2i–k were measured using three independently fabricated devices. For Figure 2h, repeatability was evaluated using two independently fabricated devices and two independently prepared phantoms, yielding three individual normalized traces. For phantom‐to‐phantom comparison, phantoms were independently prepared using the same recipe, and each device/phantom dataset was normalized to its own reference value to compare the design trend rather than absolute inter‐phantom coupling strength.

### 4.2 Impedance‐Based DTCP Model

For the impedance‐based DTCP circuit model in Figure S3, Tx‐side tissue‐coupled electrode impedance, Rx‐side tissue‐coupled electrode impedance, receiver load impedance, and tissue‐path gain/loss were measured as frequency‐dependent lumped parameters. These measured quantities were used in a simplified circuit model to estimate normalized load power as a function of frequency. Two calculation methods were used: a mean‐input model, in which the mean values of the measured parameters were used as model inputs, and a combo‐mean approach, in which load power was calculated for all 48 measured parameter combinations and then averaged. The model was used to provide a qualitative impedance‐based rationale for the favorable MHz operating range rather than to reproduce the exact measured PTE spectrum.

### 4.3 Impedance Matching Network (MN) Design

The impedance MN was designed based on in vivo impedance measurements of the electrode–tissue interface. A dummy implant incorporating identical PEDOT‐coated electrodes but no internal circuitry was used to measure the electrode impedance in vivo with a vector network analyzer (VNA). Subsequently, the input impedance from the electrodes to the ASIC terminals was characterized under the same settings. Using these two measured impedance values, an L‐type MN topology was modeled and optimized in simulation to maximize PTE. This configuration was selected because it requires only two passive components, offering the simplest structure and smallest footprint. In practice, the required inductance for impedance matching was on the order of 5 µH due to the small system capacitance and the resulting high impedance level. At 16–28 MHz, commercial 0603 or 0402 inductors of this value exhibited a low Q‐factor (Q < 5), introducing substantial loss. Reducing the inductance to achieve a higher Q‐factor shifted the matching frequency and diminished the matching effect. After iterative evaluation of component combinations, the best experimental configuration yielded a ∼30% increase in PTE as shown in Figure 2e. However, the added discrete components increased the total volume from 2.3 mm^3^ to 3.0 mm^3^, thus limiting the benefit for miniaturized systems. These trade‐offs will be revisited in future designs through optimization of system capacitance and higher levels of integration.

### 4.4 TINY Implant Fabrication and Assembly

The ASIC was fabricated using the TSMC 65 nm RF LP CMOS process with a 1P9M + Al metal stack (nine copper layers with a top aluminum layer serving as the exposed pad metal). After wafer processing, individual dies were singulated using a 15 µm‐wide diamond dicing blade, yielding chips measuring 0.6 × 0.3 × 0.3 mm^3^ (length × width × thickness). The die thickness can be further reduced by thinning in future implant iterations. The flexible substrate was a 100 µm‐thick polyimide PCB with electroless nickel immersion gold (ENIG) surface finish, featuring a 3 µm gold plating. The ASIC was picked and placed using a T‐4909‐AE manual die bonder and attached to the PCB with MG Chemicals 9310 non‐conductive epoxy, cured at 120°C for 10 min. Prior to wire bonding, both the die surface and PCB pads were cleaned sequentially with acetone and deionized water. K&S 4124 Ball Bonder was employed for gold wire bonding. Because the flexible substrate provides limited mechanical rigidity, the wedge‐bond joints were reinforced using EPO‐TEK H20E silver epoxy, cured at 120°C for 15 min. The entire assembly was then encapsulated with SYLGARD 184 PDMS (10:1 base to curing agent). After degassing for 10 min, the PDMS was applied uniformly over the ASIC and bonding wires using a micro‐pin tool, followed by curing at 150°C for 10 min. This coating procedure was repeated multiple times until sufficient encapsulation thickness was achieved to fully cover the bonded area.

The implant's Rx electrodes measured 900 × 900 µm^2^, while the stimulation electrodes were 600 × 600 µm^2^ with a 1.4 mm pitch. To reduce electrode impedance and enhance tissue contact, PEDOT electroplating was performed. The plating solution consisted of lithium perchlorate (LiClO_4_, 120 mM, 0.24 g) and EDOT monomer (30 mM, 60 µL) dissolved in 20 mL acetonitrile (ACN). A stainless‐steel wire (diameter 254 µm) served as the negative electrode, and deposition was conducted using a DC current of 100 µA for 100 s. After coating, the electrodes were rinsed sequentially with acetone and deionized water. For devices incorporating an impedance matching network, 183°C solder was used for component attachment, and the soldered area was subsequently covered with the same PDMS formulation for protection and insulation. Figure S17 shows photographs of the TINY device prototype.

### 4.5 Accelerated Aging Test

Accelerated aging was performed by immersing independently fabricated implants in standard saline at 87°C. PTE was measured before aging and after the specified soaking durations using the same characterization setup. Device 1 was measured after 1 and 7 days of soaking, corresponding to estimated equivalent aging durations of 32 and 224 days at body temperature. Device 2 was measured after 1 to 4 days, corresponding to 32 to 128 days. Device 3 was measured after 1 to 3 days, corresponding to 32 to 96 days. Detailed PTE changes are summarized in Figure S16.

### 4.6 Phantom Preparation and Benchtop Characterization

A tissue‐mimicking gel phantom was prepared by mixing 1× PBS diluted 20‐fold with 1.8% agar powder (w/v). The mixture was stirred and microwaved alternately until the solution became clear and homogeneous, ensuring complete dissolution of agar. The liquid was then refrigerated at 4°C for 2 h to allow solidification, resulting in a transparent and uniform gel phantom suitable for electrical characterization. This specific formulation is chosen for several reasons. First, using undiluted PBS would lead to unrealistic conditions, since the conductivity of muscle tissue is around 0.5 S/m [56], whereas that of standard PBS is about 11 S/m [64], far higher than physiological levels. Second, employing a purely liquid medium would result in perfect and constant contact between the phantom and the implant, leading to an overly optimistic estimation of PTE. To address these issues, PBS is diluted to reduce conductivity, and agar is added to solidify the mixture. For selected measurements requiring a more tissue‐relevant interface, including the updated frequency and length/depth characterization, a freshly excised rat‐skin layer was placed on top of the PBS‐agar phantom. The implant was positioned beneath the skin layer at the target depth in the agar. The rat‐skin layer was prepared immediately before measurement and kept hydrated with PBS during testing to reduce dehydration‐induced variability. Bulk biological tissues such as pork or ground beef were not used as the primary phantom for systematic parameter sweeps because their surface hydration and contact conditions change rapidly after air exposure, introducing uncontrolled variability. Moreover, since an LED‐based optical indication is used to assess PTE, the color and opacity of real tissue could interfere with optical detection.

All benchtop measurements were conducted using a fully battery‐powered setup to eliminate ground loops and minimize electromagnetic interference. A MHz sinusoidal signal was generated (Siglent SSG3021X), amplified (Mini‐Circuits ZHL‐20W‐13+) and then fed into a 180° –3 dB hybrid coupler (Mini‐Circuits ZAPDJ2‐5W‐521S+) to produce a balanced differential signal.

Each output arm of the coupler was connected through identical SMA coaxial cables to a bidirectional coupler (Mini‐Circuits ZFBDC26‐52HP‐S+), enabling simultaneous monitoring of forward and reflected power. During testing, a Siglent SSA3032X spectrum analyzer was used to measure both forward and reverse power from the couplers.

### 4.7 Surgery Technique

Adult male Sprague–Dawley rats (RRID: RGD_734476) were anesthetized with 2%–3% isoflurane in oxygen (500 mL min^−^^1^) and maintained at 36°C–37°C using a thermostatically controlled heating pad. The tail was grounded, and the left thigh was shaved and sterilized. A lateral skin incision was made along the left hind limb, extending from the hip to the knee, parallel to the femur. The vastus lateralis and biceps femoris muscles were gently separated to expose the sciatic nerve. A wireless stimulator was positioned with its stimulation electrodes aligned to the nerve and its power electrodes facing the vastus lateralis muscle. The skin incision was then closed with 4‐0 polypropylene sutures. The transmitter circuit and electrode fabrication were described in detail in the main text (Figure 3). After closure, two transmitter electrodes (micro‐pins) were inserted percutaneously, one near the hip and the other near the knee, approximately 22–25 mm apart. At the end of the experiment, animals were euthanized according to approved protocols. Acute stimulation experiments were performed in six rats. The carrier frequency and Tx input power were optimized for each animal; detailed conditions are summarized in Table S3. Histology: fixed in 4% paraformaldehyde (PFA) for 24 h at 4°C. Following fixation, the samples were washed three times with phosphate‐buffered saline (PBS) to remove residual fixative and then transferred to 70% ethanol (EtOH). The preserved tissues were embedded in paraffin blocks for sectioning (5 µm). Paraffin sections were stained with hematoxylin and eosin (H&E). All procedures were approved by the Institutional Animal Care and Use Committee of the University of Florida (IACUC 202500000323) and conducted in compliance with institutional and NIH guidelines. No cell lines or antibodies were used in this study.

### 4.8 Data Acquisition and Analysis

EMG recordings were obtained using TE/S46‐638 Pt/Ir subdermal needle electrodes. The signal and reference electrodes were inserted into the biceps femoris with an inter‐electrode spacing of 10 mm, and the ground electrode was placed on the rat's neck. Signals were amplified by an AM1800 recorder (bandwidth 0.5–5 kHz, gain 1000×) and digitized by an Intan recording system at a 30 kHz sampling rate. Data were extracted using MATLAB scripts. Stimulation was applied with a pulse pattern of 500 µs width at 1 Hz. Each stimulation artifact was used to synchronize the EMG responses across trials. The signals were segmented and averaged across 30 repetitions to obtain the mean EMG waveform shown in Figure 4a.

## Author Contributions

**Han Wu**: conceptualization, investigation, writing – original draft, writing – review and editing, visualization, validation, formal analysis, project administration, data curation, software, methodology. **Sultan Mahmud**: investigation, validation, methodology, data curation, formal analysis, visualization. **Anvi Singh**: validation, data curation. **Mali Halac**: investigation, validation, methodology, data curation, formal analysis, writing – review and editing, visualization. **Shriya Srinivasan**: supervision, writing – review and editing. **Erin Patrick**: supervision. **Chaojun Cheng**: validation, visualization, data curation. **Asif Iftekhar Omi**: conceptualization, investigation, writing – review and editing, visualization, software, data curation, methodology. **Jeremiasz Dados**: visualization, validation, formal analysis, data curation. **Baibhab Chatterjee**: supervision, writing – review and editing, project administration, funding acquisition. **Domenica Sofia Baez Rodas**: writing – review and editing, visualization, validation, formal analysis, data curation. **Adam Khalifa**: supervision, resources, project administration, writing – review and editing, visualization, funding acquisition. **Cameron Wallace**: software, visualization. **Anyu Jiang**: validation, visualization, data curation.

## Funding

Research reported in this publication was supported by the National Institute of Biomedical Imaging and Bioengineering of the National Institutes of Health under Award Number DP2EB037188 and National Science Foundation Career Award Number ECCS 2441610.

## Use of AI‐Assisted Technologies

During the preparation of this manuscript, the authors used AI‐assisted tools for language polishing, grammar editing, and translation support. AI tools were not used to generate or modify any images, figures, data, analyses, key arguments, or scientific conclusions. All AI‐assisted text was reviewed, edited, and verified by the authors, who take full responsibility for the final content of the manuscript.

## Conflicts of Interest

The authors declare no conflicts of interest.

## Supporting information




**Supporting File**: advs76915‐sup‐0001‐SuppMat.docx.

## Data Availability

The data that support the findings of this study are available from the corresponding author upon reasonable request.
