# Supplementary material for: Differential Tissue‐Coupled Powering for Battery‐Free Injectable Electroceuticals
Source: Adv Sci (Weinh). 2026 Aug 5;13(60):e76915. doi: 10.1002/advs.76915 (PMC13440191; doi:10.1002/advs.76915)
Supplement: Supplementary file 1 [file ADVS-13-e76915-s001.docx]

Supporting Information

**Differential Tissue-Coupled Powering for Battery-Free Injectable Electroceuticals**

This supplementary file includes:

ToC figure (55mm×50mm)

Supplementary Tables 1-3

Supplementary Figures 1-11

Supplementary Notes 1

**ToC figure (55mm×50mm)**

**ASIC**

**Electrodes**

**Table S1. Comparison with representative academic state-of-the-art wireless neural stimulator systems**

|  | Nature BME 20’[6] | Nature BME 22’ [23] | TBIOCAS 18’[8] | JNE 22’ [33] | TBIOCAS 19’ [13] | TINY |
| --- | --- | --- | --- | --- | --- | --- |
| Link | Ultrasound | ME | Ultrasound | Volume conduction | RF link | DTCP |
| Frequency (Hz) | 1.85M | 300-400k | 1.3M | 3M | 1.18G | 10-30M |
| Process (nm) | 65 | 180 | 180 | 180 | 130 | 65 |
| Pulse width (µs/s) | 392 | 1500 | 14-470 | 200 | 500 | Tunable^a^ |
| Stimulation frequency (Hz) | 0.33 | 3 | up to 60 | 2.5-3.3 | 1 and 5 | Tunable^a^ |
| Mass(mg) | 6.4 (exclude cuff) | 30 | 78 | N/R | <1 | **8** |
| Volume(mm^3^) | 4.9^b^ | 6.2 | 39 | 25.9 | 0.009 | **2.3** |
| Off-implant elements | Cuff | nothing | Storing capacitor | nothing | nothing | **nothing** |
| Portable External Transmitter | No | No | No | No | No | **Yes** |
| Rx Electrode size (mm) | 0.55×0.55 | 1×1 | Φ=0.5mm, 2mm long | Φ=0.25mm, 6 mm long | 0.25×0.08 | 0.9×0.9 |
| Phantom Model | ultrasound gel | pork | castor oil | N/R | Beef | diluted PBS + Agar |
| Injectability | No | No | No | Yes | No | **Yes** |
| Surgery Model | rat sciatic nerve | rat sciatic nerve | frog hind leg | Rabbit hindlimb | rat sciatic nerve | rat sciatic nerve |
| In vivo depth (mm) | 18 | 15 | N/R | N/R | 5 | 10 |
| In vitro depth (mm) | 48 | 20 (report 0-40) | 105 | N/R | 6.6 | 10  (report 5-20) |
| PTE (%) at the above depth | 0.7 | 0.12 | 0.04 | 0.044^c^ | 0.0019 | 0.38 |
| FoM1(max reported depth mm/volume mm^3^) | 9.8 | 6.5 | 2.7 | 0.23^d^ | 733 | **8.7** |

^a^The pulse width and stimulation frequency are programmable on the Tx side. There is no clear limit on the ASIC.

^b^Cuff 3.2 mm^3^, implant 1.7 mm^3^

^c^Calculated from 41 V 200mA input and 2mA maximum 1.8V output.

^d^Estimated from the max intramuscular depth.

**Table S2. Comparison with representative Commercial neural stimulator systems**

|  | StimRouter [39]-[41] | Curonix/Freedom PNS [42]-[44] | Nalu Neurostimulation [45]-[46] | NuStim [47]-[50] | Neuspera iSNM [51]-[54] | TINY |
| --- | --- | --- | --- | --- | --- | --- |
| Link | electric-field conduction | RF link | RF link | RF link | RF link | DTCP |
| Implanted Electronics | No | No | PCB | PCB | ASIC | **ASIC** |
| Frequency (Hz) | N/A | 915M | 40.68M power  2.4G data | 6.78M | 902-928M | 10-30M |
| Pulse width (µs/s) | 70-500 | 50-500 | 12-2000 | 40-450 | 105-945 | Tunable^a^ |
| Stimulation frequency (Hz) | 1-200 | 5-1500 | 2-1500 | 4-50 | 5-100 | Tunable^a^ |
| Implanted hardware size | L=15cm  Φ=1.2mm | Electrode Array:  L=45cm  Φ=1.35mm  Receiver:  L=47cm  Φ=0.35mm | Lead:  L=40/60cm  Φ=1.3mm  IPG:  28×11×4.9mm | L=10mm  Φ=3mm  V=71mm^3^ | L=44mm  Φ=2.3mm  V=183mm^3^ | **2.3 mm^3^** |
| Weight (g) | N/R | N/R | 80 | N/R | N/R | **0.008** |
| Portable External Transmitter | Yes | Yes | Yes | No | Yes | **Yes** |
| Rx Electrode size | 1 mm long with 1 mm spacing | 12.72 mm^2^ | 12.25 mm^2^ | 7 mm^2^ | 3 mm length with 3 mm spacing | **0.81mm^2^** |
| Injectability | Yes | No | No | Yes | Yes | **Yes** |

^a^The pulse width and stimulation frequency are programmable on the Tx side. There is no clear limit on the ASIC.

**Table S3. Summary of in vivo animal experiments and stimulation conditions.**

| **Trial number** | **Rat age (days)** | **Rat weight (g)** | **Best RF power Frequency (MHz)** | **Minimum power sent to Tx electrodes to stimulate (W)** |
| --- | --- | --- | --- | --- |
| 1 | 138 | 652 | 22 | 1 |
| 2 | 85 | 411 | 25 | 1 |
| 3 | 112 | 452 | 25 | 0.8 |
| 4 | 83 | 328 | 28 | 1.3 |
| 5 | 112 | 410 | 20 | 1.3 |
| 6 | 170 | 723 | 20 | 1 |

**Figure S1. Schematic, photo, and results of the in vitro wireless power transfer measurement setup using pork tissue. a.** A battery-powered signal generator (isolated from earth ground) was used to generate a 10 MHz RF signal, which was amplified to 26–33 dBm through a power amplifier and identical coaxial cables across all tests. The Tx electrode spacing was set to 10 mm (edge-to-edge). The implant model was placed beneath a 20-mm-thick pork tissue sample with intact skin. LED brightness was recorded using a stationary smartphone camera, ensuring that both the LED and camera remained fixed throughout the experiment. **b.** The LED did not emit any measurable brightness in the absence of tissue, whereas noticeable brightness was observed with tissue present. By comparing the measured LED brightness with the calibration database, the total received power (including ASIC consumption and LED optical output) was determined to range from 0.315 mW to 2.08 mW at 10 MHz, corresponding to a total efficiency of 0.31–0.48% for input powers of 100–447 mW (after accounting for reflection loss). Efficiency fluctuates primarily due to the rectifier, which operates more efficiently at higher input voltages. While surface coupling is feasible, stable fixation on rat skin proves challenging and limits repeatability; therefore, pin electrodes are adopted in the main experiments to ensure repeatable conditions. Future designs aim to improve contact stability and re-enable surface electrodes.

**Figure S2. Voltage limiter circuits in ASIC.** The limiter consists of three cascaded MOSFET stages that progressively conduct to clamp excessive rectified voltage $V_{\mathrm{rec}}$. The first stage forms a high-impedance voltage divider that consumes only nW-level leakage within the normal operating range. When $V_{\mathrm{rec}}$ rises above the designed threshold, the voltage across the first-stage PMOS is large enough to turn on the second-stage PMOS, increasing the drain current to limit the voltage. If $V_{\mathrm{rec}}$ further increases, the third-stage NMOS conducts strongly, causing an exponential rise in drain current to tightly clamp $V_{\mathrm{rec}}$. This passive limiter protects both the implant circuitry and tissue, since $V_{\mathrm{rec}}$and ground are directly connected to the stimulation electrodes.

**Figure S3. Impedance-based DTCP circuit model for explaining the favorable MHz operating window.** Measured frequency-dependent components used in the simplified DTCP model, including the Tx-side tissue-coupled electrode impedance Z_Elec_Tx_, Rx-side tissue-coupled electrode impedance Z_Elec_Rx_, receiver load (ASIC) impedance Z_Load_, and tissue-path gain/loss. The analyzed frequency range is from 100 kHz to 45 MHz. The number of repeated measurements used for each quantity is indicated in the corresponding panel. The shaded regions show mean ± SD, and the red curves show the mean values. The equivalent circuit used to estimate receiver load power is shown in the inset of the bottom panel. The normalized load-power trend was calculated using two approaches. In the “mean-input model,” the mean values of Z_Elec_Tx_, Z_Elec_Rx_, Z_Load_, and tissue-path gain/loss were used as the circuit inputs to estimate the load power at each frequency. In the “combo mean” approach, load power was first calculated for all 48 measured combinations of input parameters, and the resulting load-power curves were then averaged. Both approaches show low estimated load power at low frequencies, improved power delivery in the MHz range, and a clear reduction above approximately 40 MHz, supporting a bounded favorable operating window rather than a monotonic improvement with frequency. Because this simplified model uses experimentally measured lumped impedance and tissue-path gain/loss parameters rather than full field-level modeling, it is intended to provide a qualitative impedance-based rationale for the favorable MHz operating range, rather than to reproduce the exact PTE spectrum or predict the precise optimum frequency.

**Figure S4. Device-to-device repeatability of Figure 2g.** Individual normalized PTE curves from four independently fabricated devices measured at dRx = 15 mm and 10 mm implantation depth. Each curve was normalized to its own maximum PTE. The consistent optimum near dTx = 18 mm supports the reproducibility of the Tx-electrode spacing design rule.

**Figure S5. Electric-field distribution underlying optimal transmitter spacing.** Finite-element simulations (HFSS) of the electric-field magnitude for two representative spacing ratios: (a) $d_{RX}=35$mm and $d_{TX}=36$mm; (b) $d_{RX}=17$mm and $d_{TX}=18$mm. The receiver electrodes are placed at a depth of 10 mm. The dominant potential drop is concentrated in the region between the transmitter electrodes, whereas the local field gradient is reduced near each transmitter due to boundary-condition clamping. The superimposed $\mathbf{E}$-field vectors predominantly traverse the inter-electrode region (arrows oriented from one transmitter electrode toward the other), corroborating that the strongest field propagation and thus the principal voltage drop occurs between the transmitter pair rather than immediately adjacent to each electrode. Because the harvested voltage equals the line integral of the electric field over $d_{RX}$ ($V_{RX}=\int_{\mathcal{l}} \mathbf{E}\cdot d\mathcal{l}$), choosing $d_{TX}$slightly larger than $d_{RX}$ensures that the receiver pair spans the high-gradient region, maximizing the differential voltage and enhancing power transfer efficiency. Excessively large $d_{TX}$increases tissue losses and reduces effective field coupling.

 **Figure S6. Repeatability of Figure 2h across devices and phantoms.** Individual normalized PTE traces for the implant-length optimization in Figure 2h. This dataset includes two independently fabricated device groups and two independently prepared phantoms using the same recipe: Device group 1 was measured in Phantom A and Phantom B, and Device group 2 was measured in Phantom A. For each implantation depth and receiver length dRx, the Tx–Rx relative position was optimized, and the frequency was swept to extract the maximum PTE. For each device/phantom dataset, all extracted PTE values were normalized to the maximum value within that dataset. Although this dataset is not a statistical n = 3 device comparison, the three individual normalized traces consistently show increasing PTE with longer dRx and gradual saturation at larger dRx, supporting that the receiver-length design trend is not specific to a single implant or phantom preparation.

**Figure S7. Simulation–measurement comparison for receiver-length scaling related to Figure 2h.** Normalized PTE as a function of the receiver electrode spacing/implant length, $d_{\mathrm{Rx}}$, at depth =15 mm. For each receiver length, the transmitter position was independently optimized to maximize the achievable PTE, rather than being fixed. Both simulation and measurement show a monotonic increase in PTE with increasing $d_{\mathrm{Rx}}$, confirming that DTCP benefits from extending the receiver electrode spacing while maintaining a small injectable cross-section. The measured trend agrees well with simulation, supporting the validity of the DTCP model and the receiver-length scaling behavior. PTE values are normalized to the simulated PTE at the maximum tested implant length of 35 mm. This result provides additional validation of the DTCP receiver-length scaling principle used in Figure 2h.

**Figure S8. Device-to-device repeatability of the depth-dependent PTE trend in Figure 2i.** Individual normalized PTE curves from three independently fabricated devices with the same receiver electrode spacing of dRx = 15 mm. For each device, PTE was normalized to its value at 5 mm implantation depth. All devices show a consistent decrease in normalized PTE with increasing depth, supporting the reproducibility of the depth-dependent attenuation trend in Figure 2i.

**Figure S9. Device-to-device repeatability of the rotation-dependent PTE trend in Figure 2j.** Individual normalized PTE curves from three independently fabricated devices measured under rotation along the implant longitudinal axis. For each device, PTE was normalized to its value at 0° rotation. All devices show only a moderate reduction in normalized PTE over 0°–90° rotation, supporting the rotation tolerance of DTCP shown in Figure 2j.

**Figure S10. Device-to-device repeatability of the tilted-implant PTE trend in Figure 2k.** Individual normalized PTE–frequency sweeps from three independently fabricated devices under the three tilted implantation conditions defined in Figure 2k. For each device, the peak PTE of each case was extracted from the frequency sweep, and the extracted peak values were normalized to the Case 3 peak value within the same device. Although the peak frequency varies slightly among devices, all devices show the same relative trend among the three cases, supporting the tilt-tolerance analysis in Figure 2k.

**Figure S11. Simulated averaged specific absorption rate (SAR) distribution under continuous-wave (CW) transmission.** Finite-element simulations (HFSS) of SAR averaged over 10 g of tissue, in heterogeneous tissue domains under 1 W input power at 25 MHz: (a) Micro-pin transmitter electrodes corresponding to the rat measurement configuration, simulated in an agar tissue-mimicking medium ($\varepsilon_{r}=91$, $\sigma=0.5\text{S/m}$). (b) Surface electrode configuration intended for human application, simulated using frequency-dependent dielectric properties from the Gabriel tissue model. In both cases, energy absorption is primarily localized at the electrode–tissue interface, with rapid attenuation into surrounding tissue layers, which is consistent with confinement of the dominant electric-field path to the inter-electrode region. No significant deep-tissue hot spots are observed, indicating confined and spatially limited thermal loading. Peak averaged SAR values are $0.819 Wkg⁻¹\text{ }$in (a) and $0.966\text{ Wkg⁻¹}$ in (b). These values remain below ICNIRP localized exposure limits (2 W kg⁻¹ for head/torso and 4 W kg⁻¹ for limbs) and therefore fall within established safety limits at 1 W excitation.

**Time-averaged SAR under pulsed transmission**

The simulated SAR of 0.8 W/kg (as an example) corresponds to continuous-wave (CW) operation with a constant transmitter power of 1 W. Under the common assumption that SAR scales linearly with time-averaged deposited power (i.e., field distribution and tissue properties are unchanged), the time-averaged SAR for a pulsed waveform can be obtained by scaling the CW SAR by the duty cycle:

$$\mathrm{SAR}_{avg}\approx\mathrm{SAR}_{CW}\cdot D$$

where $D$is the fraction of time the RF carrier is “ON” within one repetition period.

For a stimulation duty cycle of 500 µs per second (i.e., total RF “ON” time $t_{on}=500\text{ }\mu s$within $T=1\text{ }s$):

$$D=\frac{t_{on}}{T}=\frac{500\times{10}^{-6}}{1}=5\times{10}^{-4}$$

Thus, the time-averaged SAR becomes:

$$\mathrm{SAR}_{avg}\approx0.8 W/kg\times5\times{10}^{-4}=0.4 mW/\mathrm{kg}$$

Note: This scaling assumes the peak RF power during each pulse remains 1 W (same as the CW simulation condition) and that the coupling/matching does not change significantly between CW and pulsed operation. The same scaling is applicable to all the CW SAR values. Also, both CW and pulsed values remain below limits in all cases.

**Figure S12. In vivo EMG signals with negative control.** Wireless stimulation evokes repeatable EMG signals at 1 Hz. A representative EMG trace (top) and the averaged response (bottom) show time-locked muscle activation synchronized with each stimulus pulse (500 μs width). A negative-control condition, acquired with the external Tx waveform applied but the implant removed, shows no corresponding EMG.

**Figure S13. a. Schematic and model parameters for a thread-like stimulator in a monofascicular nerve, showing electrode placement and tissue properties used for simulation.** A finite element model was constructed in COMSOL Multiphysics (COMSOL Inc., USA) to compute the electric potential field generated by the implant under a fixed 1 mA input current with an electrode-to-fascicle distance of 100 µm. Two key parameters were varied: electrode pitch (100 µm–1 mm) and electrode surface area (0.0025–0.09 mm²), yielding 24 geometric configurations. Neural activation was then simulated in NEURON using the McIntyre–Richardson–Grill (MRG) axon model tuned for a 6 µm rat tibial fiber. A cathodic-first symmetric biphasic pulse (200 µs width) was applied, and the threshold amplitude was defined as the minimum stimulus current that elicited a propagating action potential. **b. Simulated activation thresholds for the neural fiber as a function of electrode pitch and size.** Both increasing electrode surface area and interelectrode pitch reduced the stimulation threshold. A pitch of 1 mm combined with a 300 µm² electrode surface achieved the lowest threshold of 0.535 mA for the modeled 6 µm fiber. Unlike conventional implants, the thread-like DTCP device can accommodate large electrode pitches, enabling more energy-efficient stimulation without sacrificing design flexibility.

**Figure S14. Electrochemical Impedance Spectroscopy (EIS) model of the PEDOT-coated stimulation electrodes.** EIS was performed using an Ivium system to characterize the stimulation electrode impedance from 1 Hz to 100 kHz. The stimulation electrode had a geometric area of 0.6 mm × 0.6 mm and was coated with PEDOT to lower the electrode–electrolyte interface impedance. The measured impedance magnitude and phase were fitted using a three-stage parallel RC equivalent-circuit model, which captured the frequency-dependent electrode behavior with good fitting accuracy. The fitted equivalent-circuit model was subsequently imported into Cadence and co-simulated with the ASIC to evaluate the electrode-loaded stimulation performance. The resulting simulation was used to determine the maximum stimulation current deliverable by the system, as described in Section 2.5.

**Figure S15. a. Photographs of a longitudinal H&E section.** The target nerve region on day 20 shows preserved nerve boundary morphology without overt fibrotic capsule formation or neutrophil-rich inflammatory infiltrate at the epineurium. **b. Fibrosis & neutrophil presence.** No evidence of fibrosis or neutrophilic infiltration is detected on the target nerves. Note that the implant anchors onto the nearby muscles. Therefore, the lack of fibrosis or neutrophilic infiltration on the target nerve is expected.

**Figure S16. Accelerated aging test of injectable implants under elevated-temperature saline soaking.** Three independently fabricated implants were immersed in standard saline at 87 °C to evaluate accelerated aging behavior. Device 1 was characterized before aging, after 1 day, and after 7 days of accelerated aging, corresponding to estimated equivalent aging durations of 32 days and 224 days at body temperature, respectively. Device 2 was characterized before aging, after 1 day, and after 4 days of accelerated aging, corresponding to estimated equivalent aging durations of 32 days and 128 days. Device 3 was characterized before aging, after 1 day, and after 3 days of accelerated aging, corresponding to estimated equivalent aging durations of 32 days and 96 days. Across the tested devices, PTE remained measurable after accelerated aging, and the additional independent devices showed comparable frequency-dependent profiles with smaller degradation over their tested aging durations. These results indicate gradual aging-related PTE degradation rather than abrupt loss of wireless power reception under accelerated saline aging conditions. Under the tested operating condition, the observed aging-related PTE reduction would reduce the available power received but would not eliminate the stimulation margin, because the loss can be compensated by a modest increase in Tx input power while remaining below the measured safety limit discussed in Section 2.4.

**Figure S17. Photographs of one TINY device sample showing the stimulation electrodes’ side and a magnified view of the ASIC assembly region.** Top: the stimulation side showing PEDOT-coated gold stimulation electrodes patterned on a flexible polyimide substrate. Bottom: the opposite side showing a magnified view of the bonded ASIC chip. After the ASIC was fixed with high-temperature glue, wire bonds were first made to 3-µm-thick gold pads on the flex PCB. Because the mechanical reliability of wire bonds on flexible substrates is relatively low, silver epoxy was subsequently applied to reinforce both the electrical and mechanical connections. Finally, PDMS was used to coat the whole assembly.

**Note S1**

According to IEEE C95.1 Table 14, the human contact current limit in the 3–30 MHz range follows

$$I_{\max}(f)=16.7{(\frac{f}{3})}^{0.3}\text{ mA},3\leq f\leq30\text{ MHz}.$$

Substituting $f=25$MHz gives $I_{\max}=16.7(25/3)^{0.3}=31.5$mA.

For comparison, the electrode–tissue impedance measured on the rat hindlimb is $R=325\text{–}362\text{ }$and $C=27.2\text{–}38.6\text{ pF}$. With 28 dBm available from a 50 Ω source and a stimulation duty of 500 µs / s, the maximum resulting 1-s-RMS current is only $\approx0.61$mA, well below the limit.
